# Small-molecule targeted therapies induce dependence on DNA double-strand break repair in residual tumor cells

**DOI:** 10.1126/scitranslmed.abc7480

**Published:** 2022-03-30

**Authors:** Moiez Ali, Min Lu, Hazel Xiaohui Ang, Ryan S. Soderquist, Christine E. Eyler, Haley M. Hutchinson, Carolyn Glass, Christopher F. Bassil, Omar M. Lopez, D. Lucas Kerr, Christina J. Falcon, Helena A. Yu, Aaron N. Hata, Collin M. Blakely, Caroline E. McCoach, Trever G. Bivona, Kris C. Wood

**Affiliations:** 1 Department of Pharmacology and Cancer Biology and Duke Cancer Institute, Duke University, Durham, NC 27710, USA; 2 Department of Pathology, Duke University, Durham, NC 27710, USA; 3 Department of Medicine and Helen Diller Family Comprehensive Cancer Center, University of California, San Francisco, San Francisco, CA 94143, USA; 4 Thoracic Oncology Service, Division of Solid Tumor Oncology, Department of Medicine, Memorial Sloan Kettering Cancer Center, Weill Cornell Medical College, New York, NY 10065, USA; 5 Massachusetts General Hospital Cancer Center and Harvard Medical School, Charlestown, MA 02129, USA

## Abstract

Residual cancer cells that survive upfront drug treatments act as a reservoir from which eventual resistant disease emerges. Although there is great interest in therapeutically targeting residual cells, efforts to do so are hampered by our limited knowledge of the vulnerabilities existing in this cell state. Here, we report that diverse oncogene-targeted therapies, including inhibitors of epidermal growth factor receptor (EGFR), anaplastic lymphoma kinase (ALK), KRAS, and BRAF, induce DNA double strand breaks and consequently, ataxia-telangiectasia mutated (ATM)-dependent DNA repair in oncogene-matched residual tumor cells. This DNA damage response, observed in cell lines, mouse xenograft models, and human patients, is driven by a pathway involving the activation of caspases 3 and 7 and the downstream caspase-activated deoxyribonuclease (CAD). CAD is, in turn, activated through caspase-mediated degradation of its endogenous inhibitor, ICAD. In models of *EGFR* mutant non-small cell lung cancer (NSCLC), tumor cells that survive treatment with small-molecule EGFR-targeted therapies are thus synthetically dependent on ATM, and combined treatment with an ATM kinase inhibitor eradicates these cells in vivo. This led to more penetrant and durable responses in *EGFR* mutant NSCLC mouse xenograft models, including those derived from both established cell lines and patient tumors. Last, we found that rare patients with *EGFR* mutant NSCLC harboring co-occurring, loss-of-function mutations in *ATM* exhibit extended progression-free survival on first generation EGFR inhibitor therapy relative to patients with *EGFR* mutant NSCLC lacking deleterious *ATM* mutations. Together, these findings establish a rationale for the mechanism-based integration of ATM inhibitors alongside existing targeted therapies.

## One Sentence Summary

Combined treatment with targeted therapies plus ATM inhibitors may lead to more penetrant and durable therapeutic responses.

## Introduction

Oncogene targeted therapies have the potential to selectively eradicate tumor cells while sparing healthy tissues, a notion supported by evidence of remarkable activity in a subset of patients with cancer. For this reason, a number of targeted therapies, including EGFR inhibitors in *EGFR* mutant NSCLC, anaplastic lymphoma kinase (ALK) inhibitors in *ALK*-rearranged NSCLC, BRAF/MEK (mitogen-activated or extracellular signal-regulated protein kinase kinase) inhibitors in *BRAF* mutant melanomas, and tropomyosin receptor kinase (TRK) inhibitors in neurotrophic tyrosine receptor kinase (*NTRK*) fusion-positive tumors have become mainstays of clinical treatment, and many additional targeted therapies are now advancing through preclinical and clinical development (1–3). Unfortunately, it is also now well established that the depth and duration of responses to these agents are limited in patients with advanced disease, as most patients progress on the timescale of months. At that point, treatment options become limited, as many mechanisms of resistance are either unknown or cannot be pharmacologically targeted and patients often simultaneously harbor multiple distinct resistance mechanisms (4, 5). This challenging reality underscores the importance of identifying more effective strategies to improve the upfront depth and duration of response to targeted therapies (6, 7).

Extensive studies have examined the interplay between DNA damaging chemotherapies or radiation therapy and resultant cellular DNA damage and repair processes. By contrast, we know relatively little about the impact of oncogene targeted therapies on these processes. Three recent studies demonstrated that inhibitors of the EGFR/RAF/MEK/ERK pathway cause transcriptional suppression of key genes involved in homologous recombination (HR) and mismatch repair (MMR) (8–10). Similarly, another pair of recent studies showed that PI3K inhibition can also suppress HR, potentially through modulation of extracellular signal-regulated kinase (ERK) activity (11, 12). Thus, targeted therapy-induced suppression of DNA repair processes such as HR can lead to a “BRCA-like” state in cancer cells that sensitizes them to poly (ADP-ribose) polymerase (PARP) inhibitors or agents like histone deacetylase 3 (HDAC3) inhibitors that can suppress expression of non-homologous end joining (NHEJ) genes (8, 9, 11, 12). Supporting these observations, a recent Phase I clinical trial indicated that the combination of the phosphatidylinositol 3-kinase alpha (PI3Kα)-specific inhibitor alpelisib and the PARP inhibitor olaparib yielded encouraging activity in patients with epithelial ovarian cancer (13).

Here, we report that targeted therapies induce DNA double stranded breaks (DSBs) and consequent DSB repair in surviving cancer cells through a pathway involving the activation of executioner caspases 3 and 7 and the downstream endonuclease CAD. Consequently, targeted therapy treatments create a synthetic dependence on the ATM kinase, a central coordinator of DSB repair. Combining oncogene targeted therapies with ATM inhibitors thus eradicates residual tumor cells that would otherwise survive treatment, leading to more penetrant and durable therapeutic responses in cellular and animal models. Consistent with these observations, we find that in patients with *EGFR* mutant NSCLC, ATM activation is observed in tumors treated with EGFR inhibitor therapy, and progression-free survival is extended when tumors harbor co-occurring *ATM* loss-of-function mutations. This work thus sets the stage for clinical studies investigating the integration of ATM inhibitors alongside existing targeted therapies.

## Results

### DNA damage is observed in cancer cells surviving targeted therapy treatments

**Figure 1:**
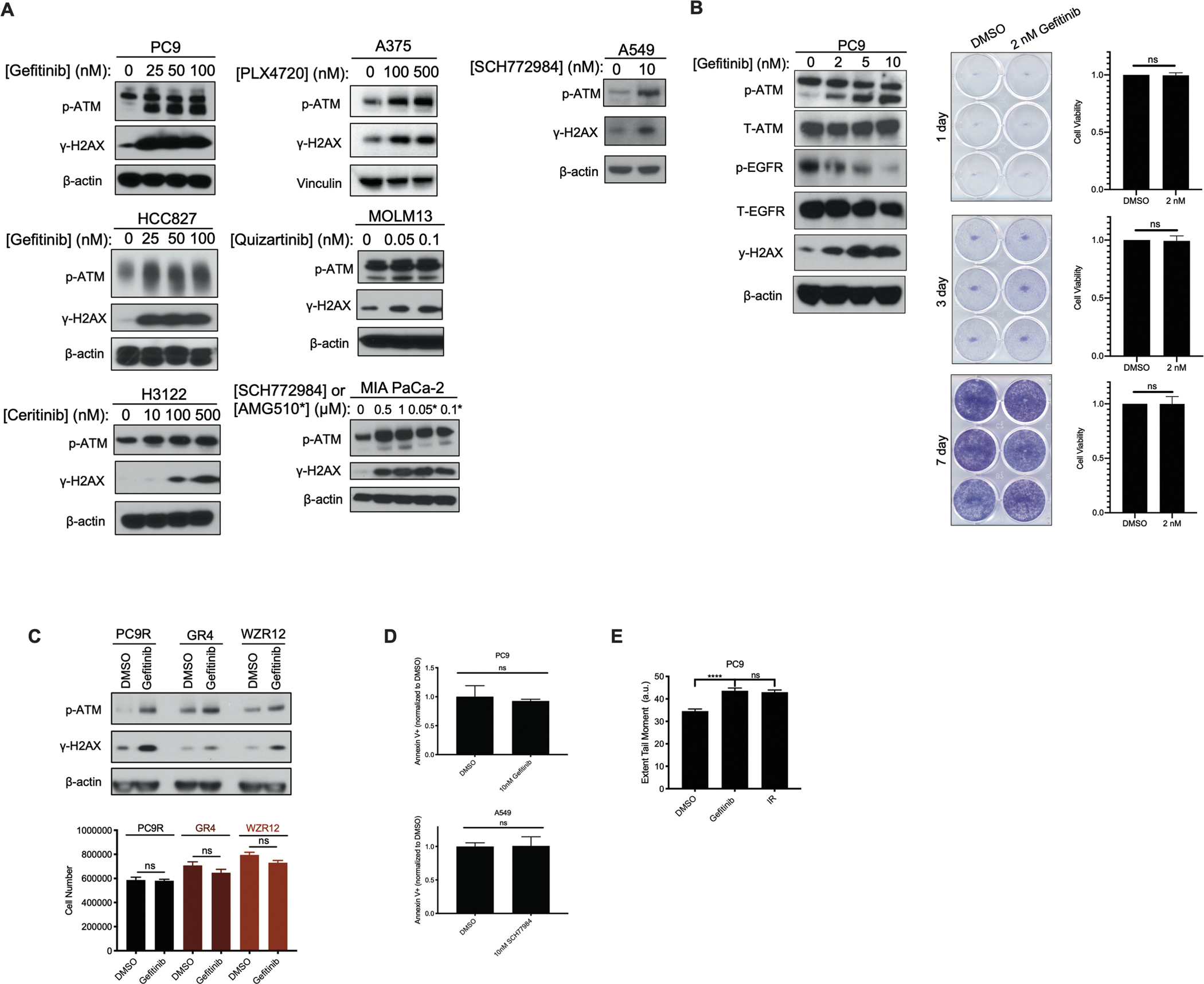
DNA damage responses in cells treated with targeted therapies. **A**. Immunoblot of NSCLC, melanoma, AML, and pancreatic cancer cell lines following 24h drug treatment with increasing concentrations of cognate targeted therapies, probing for marks of DSBs, including p-ATM at S1981 and γ-H2AX. PC9 and HCC827 are *EGFR* mutant NSCLC; H3122 is *ALK* rearranged NSCLC; A549 is *KRAS(G12S)* mutant NSCLC; A375 is *BRAF* mutant melanoma; MOLM13 is *FLT-3* mutant AML; and MIA PaCa-2 is *KRAS(G12C)* mutant pancreatic cancer. Gefitinib is an inhibitor of EGFR; ceritinib is an inhibitor of ALK; SCH772984 is an inhibitor of ERK1/2; PLX4720 is an inhibitor of BRAF; quizartinib is an inhibitor of FLT-3; and AMG510 is an inhibitor of KRAS(G12C). **B**. Immunoblot of PC9 cells treated with the indicated doses of gefitinib for 24h, alongside cell viability measures as assessed by crystal violet staining of cells in clonogenic assay plates or Cell Titer Glo (CTG) following treatment with vehicle (DMSO) or gefitinib for the indicated periods of time. N=3 for all cell viability experiments, where the mean ± S.E.M is plotted. *P* values were determined using unpaired, two-tailed Student’s *t*-tests. **C**. Cell counts following 24h of 100nM gefitinib drug exposure in drug-resistant NSCLC cells, alongside immunoblots of the corresponding drug-treated populations of cells. N=3 for cell count experiments, where the mean ± S.E.M is plotted. *P* values were determined using unpaired, two-tailed Student’s *t*-tests. **D**. Annexin V^+^ staining (normalized to DMSO vehicle control) in drug treated populations of NSCLC cell lines. N=3 for the Annexin V^+^ staining experiments, where the mean ± S.E.M is plotted. *P* values were determined using unpaired, two-tailed Student’s *t*-tests. **E**. Bar graph quantification of extent tail moment (a.u.) from neutral comet assay performed in PC9 cells, following treatment with 100 nM gefitinib for 24h (IR dose: 10 Gy). N=503 for DMSO treatment, N=704 for gefitinib treatment and N=645 for IR treatment. The mean ± S.E.M is plotted. *P* values were determined using one-way ANOVA with Tukey’s post hoc test. **** refers to P<0.0001.

To examine whether treatment of oncogene-driven cancer cells with targeted therapies results in DNA damage and subsequent activation of DNA damage response (DDR) pathways, we began by using a panel of oncogene-driven cancer cell lines responsive to their cognate targeted therapies. Treatment of *EGFR* mutant NSCLC, *ALK* rearranged NSCLC, *KRAS* (Kirsten rat sarcoma viral oncogene homolog) mutant NSCLC, *BRAF* mutant melanoma, *FLT3* (fms-like tyrosine kinase 3) mutant acute myeloid leukemia (AML), and *KRAS* mutant pancreatic ductal adenocarcinoma cell lines with increasing doses of their cognate targeted therapies for 24h led to increased amounts of autophosphorylated ATM at serine residue 1981 (S1981), a site required for activation of the downstream DNA damage response pathway, and γ-H2AX, a canonical marker of DSBs (Fig. 1A and fig. S1A) (PC9, p-ATM, *P* = 0.001 and γ-H2AX, *P* = 0.004; A375, p-ATM, *P* = 0.08 and γ-H2AX, *P* = 0.02; HCC827, p-ATM, *P* = 0.006 and γ-H2AX, *P* = 0.002; MOLM13, p-ATM, *P* = 0.06 and γ-H2AX, *P* = 0.05; H3122, p-ATM, *P* = 0.005 and γ-H2AX, *P* = 0.07; Mia PaCa-2, p-ATM, *P* = 0.08 and γ-H2AX, *P* = 0.004). To determine whether the observed DNA damage response was a trivial consequence of drug-induced cell death, a subset of these cell lines were treated with low dose targeted therapies and cell viability was assessed following up to seven days of drug exposure. At drug doses that did not impact cell viability, γ-H2AX induction was nevertheless observed (Fig. 1B and fig. S1B). Consistent with this result, we also observed gefitinib-induced increases in phosphorylated ATM and γ-H2AX in a panel of three independently derived gefitinib-resistant, *EGFR* mutant NSCLC cell lines whose growth was not affected by gefitinib treatment (Fig. 1C and fig. S1C) (p-ATM, *P* = 0.09 and γ-H2AX, *P* = 0.08). These gefitinib-resistant cell lines include PC9-R, a pooled population of resistant cells derived by stepwise selection with gefitinib; PC9-WZR12, a clonally derived line with acquired resistance to both gefitinib and the third-generation irreversible EGFR inhibitor WZ4002 harboring both an *EGFR*^*T790M*^ mutation and a *MAPK1* amplification; and PC9-GR4, harboring an *EGFR*^*T790M*^ mutation (14)). Consistent with these results, we observed no evidence of Annexin V+ staining in PC9 and A549 cells treated with cognate targeted therapies at doses and a timescale on which a DNA damage response is usually observed (Fig. 1D). Finally, a neutral comet assay directly detected the presence of DSBs following a 24h targeted therapy treatment, as evidenced by an increased extent olive tail moment, suggesting that the observed ATM activation occurred because of DNA damage (Fig. 1E). Together, these data demonstrate that cancer cells surviving treatment with matched targeted therapies exhibit DNA double strand breaks and consequent ATM activation.

### Targeted therapy treatment activates ATM kinase through mitochondrially stimulated caspase signaling

**Figure 2:**
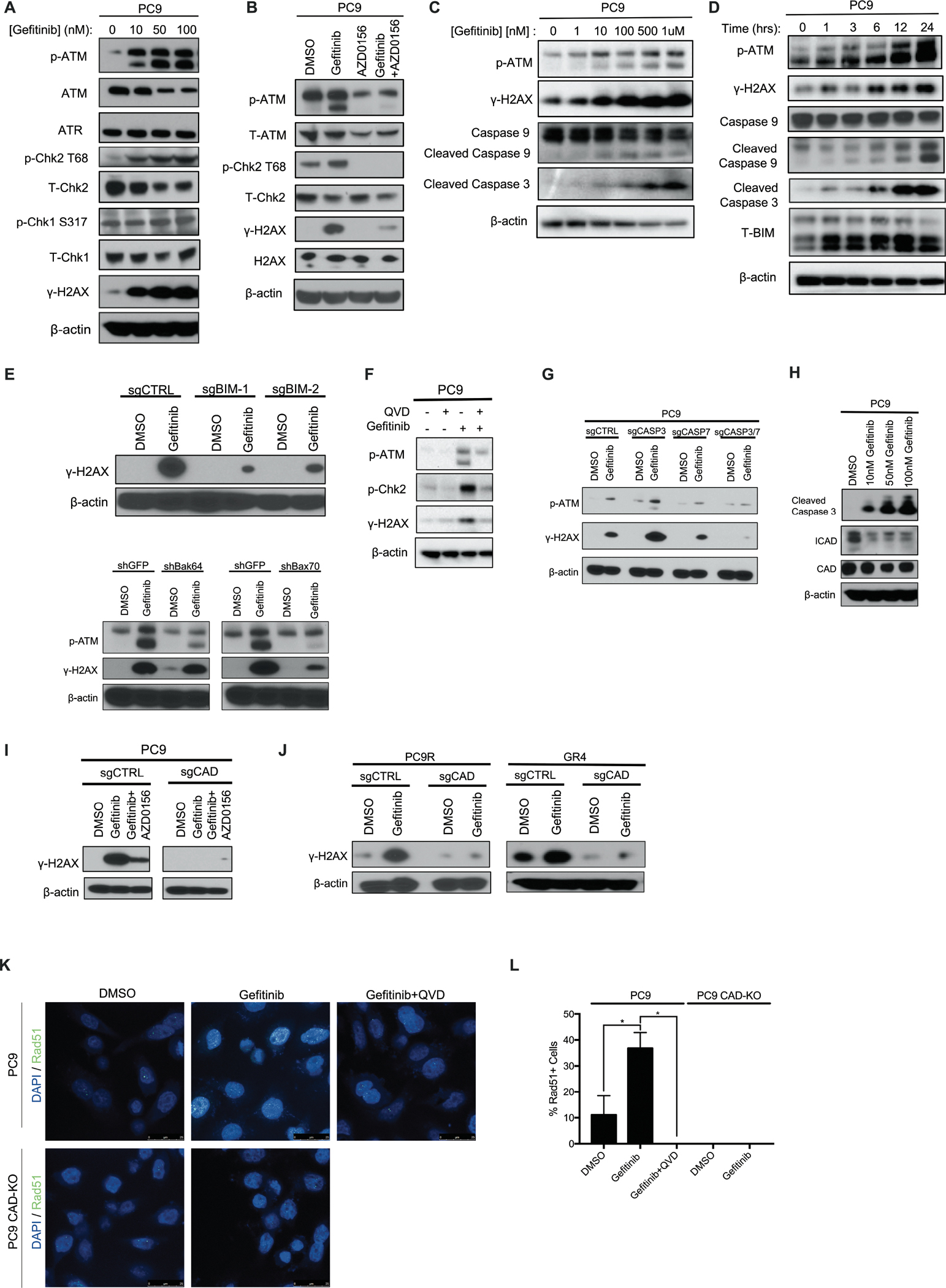
Characterization of EGFR inhibitor-induced ATM pathway activation. **A**. Immunoblotting of various DNA damage response markers in PC9 cells following 24h treatment with gefitinib at the indicated doses. **B**. Immunoblot of PC9 cells following treatment with the EGFR inhibitor gefitinib (100 nM), AZD0156 (pharmacological inhibitor of ATM, 1.5 μM), or the combination of both EGFRi and ATMi for 24h. **C**. Immunoblot of PC9 cells treated with increasing concentrations of gefitinib for 24h. **D**. Immunoblot following treatment of PC9 cells with 100nM gefitinib for the indicated lengths of time. **E**. Immunoblot of 24h, 100 nM gefitinib-treated cells following CRISPR/Cas9-mediated knockout of BIM or RNAi-mediated short hairpin (shRNA) knockdown of BAK/BAX in PC9 cells. **F**. Immunoblot of PC9 cells treated with pan-caspase inhibitor Q-VD-OPh (2 μM), gefitinib (500 nM), or the combination for 24h. **G**. Immunoblot of PC9 cells following CRISPR/Cas9-mediated knockout of caspase 3, 7, or 3+7, post 24h, 100 nM gefitinib treatment. **H**. Immunoblot of 24h gefitinib-treated PC9 cells, revealing ICAD loss. **I**. Immunoblot of 24h, 100 nM gefitinib-treated cells following CRISPR/Cas9-mediated knockdown of CAD in PC9 cells. **J**. Immunoblot of 24h, 100 nM gefitinib-treated cells following CRISPR/Cas9-mediated knockout of CAD in EGFR inhibitor-resistant cells. **K**. Confocal microscopy images of Rad51 loading assay in PC9 cells following treatment with 100 nM gefitinib, Q-VD-OPh (2 μM) or the combination for 24h with and without the presence of CAD. **L**. Bar graph quantification of images in (K). N=3 for all groups presented, where the mean ± S.E.M is plotted. *P* values were determined using one-way ANOVA with Tukey’s post hoc test. * refers to P<0.05.

To better characterize the targeted therapy-induced DDR, we measured the activation state of ATM and ATR (ataxia telangiectasia and Rad3-related protein). ATM and ATR are members of the class-IV phosphoinositide 3-kinase (PI3K)-related kinase (PIKK) family of proteins which serve as key regulators of the DNA damage response, as well as downstream signaling pathways. Although no changes in ATR or phosphorylated Chk1 (S317), a marker of ATR activation, were seen in PC9 cells treated with gefitinib, phosphorylation of ATM and its substrate Chk2 (T68) were observed in two different EGFR inhibitor-sensitive cell lines (PC9, Fig. 2A, and HCC827, fig. S2A). Additionally, similar activation of ATM and downstream effectors was observed in PC9 cells treated with the structurally distinct third-generation EGFR inhibitor osimertinib (fig. S2B). This effect was reversible (fig. S2C) and could be phenocopied via short hairpin RNA (shRNA)-mediated EGFR knockdown (fig. S2D). As ATM appears to be the major DDR pathway activated following treatment with targeted therapies, we treated PC9 cells with AZD0156, a potent, selective, and orally bioavailable ATM inhibitor, alone or in combination with EGFR blockade. This treatment confirmed that combination of an ATM inhibitor and EGFR blockade abrogated both induction of γ-H2AX and activation of the DSB repair pathway (Fig. 2B). At higher doses of gefitinib, certain proteins involved in homologous recombination were downregulated, such as EXO1, BRCA1, BRCA2, consistent with recently described findings (10) (fig. S2E). Together, these data demonstrate that EGFR inhibition leads to a DSB repair response coordinated by ATM.

During these studies, we noticed that treatment with increasing doses of gefitinib led to a dose- and time-dependent activation of ATM and γ-H2AX along with cleavage of both initiator caspase 9 and executioner caspase 3 (Fig. 2, C and D). A recent study demonstrated that activation of intrinsic pathway caspases can cause the formation of DSBs and subsequent activation of ATM, even when those caspases are activated at sub-lethal amounts (15). Thus, we hypothesized that caspase activation, occurring downstream of BIM and BAK/BAX activation and the resultant mitochondrial outer membrane permeabilization (MOMP) (16) (Fig. 2D), could be responsible for the observed DSB formation and ATM activation in cells surviving treatment with targeted therapies. Indeed, clustered regularly interspaced short palindromic repeats (CRISPR)-mediated knockout of BIM and RNAi-mediated knockdown of BAX (but not BAK) abrogated targeted therapy induced ATM and γ-H2AX activation in PC9 cells (Fig. 2E and fig. S2F–G), which is consistent with previous studies showing the preferential activation of BAX via BIM (17). To further test this hypothesis, we used the pan-caspase inhibitor Q-VD-OPh (Quinoline-Val-Asp-Difluorophenoxymethylketone), which was sufficient to abrogate the activation of ATM observed with EGFR inhibition alone (Fig. 2F). More specifically, our results point to the canonical executioner caspases 3 and 7 as drivers of the DDR, as CRISPR/Cas9-mediated knockout of both these proteins at the same time abrogated both ATM activation and γ-H2AX formation in PC9 cells treated with gefitinib (Fig. 2G and fig. S2H), a mechanism we also validated in an additional model of *EGFR* mutant NSCLC (fig. S2H–I).

CAD is a key enzyme activated by caspases 3 and 7 that has previously been shown to mediate the formation of DSBs (15). Although CAD expression was relatively unchanged following treatment of PC9 cells with gefitinib, expression of ICAD, an endogenous inhibitor of CAD that is directly cleaved by executioner caspases (15), was dramatically reduced following treatment at the same doses (Fig. 2H and fig. S2B) (*P* = 0.003). This loss of ICAD was abrogated following CRISPR/Cas9-mediated knockout of caspases 3 and 7 (fig. S2J) and was due to a decreased half-life of the protein following targeted therapy treatment (*P* = 0.03). Consistent with caspase-mediated degradation, this coincided with increased amounts of active cleaved caspase 3 and active ATM (fig. S2K). Cleavage and degradation of ICAD is expected to result in CAD activation and consequent DSB formation and ATM activation. To test this model, we knocked out CAD using CRISPR/Cas9. In the absence of CAD, we no longer observed a targeted therapy-mediated induction of γ-H2AX expression or Rad51 foci, another marker of DSB repair, within PC9 cells (Fig. 2I–2L and fig. S2L). The presence of CAD was also shown to be crucial for DSB formation in two independently-derived EGFR inhibitor-resistant populations (Fig. 2J and fig. S2L). Despite CAD knockout in PC9 cells, which resulted in abrogation of DDR activation following treatment with targeted therapy, we still observed cleavage of caspase 3 and loss of ICAD, underscoring the notion that caspase activation and ICAD loss function upstream of CAD activation (fig. S2M). Additionally, CAD knockout reduced the drug-induced extent tail moment observed in the neutral comet assay to assess DNA DSBs in PC9 cells, supporting its proposed role in inducing the formation of DNA DSBs following targeted therapy treatment (fig. S2N). Lastly, consistent with the hypothesis that ATM activity is required for the repair of targeted therapy-induced DNA damage, we observed increased Rad51 staining in cells treated with the EGFR inhibitor gefitinib and AZD0156 relative to vehicle or gefitinib alone (fig. S2O–P).

### Cancer cells surviving EGFR inhibitor therapy require ATM

**Figure 3:**
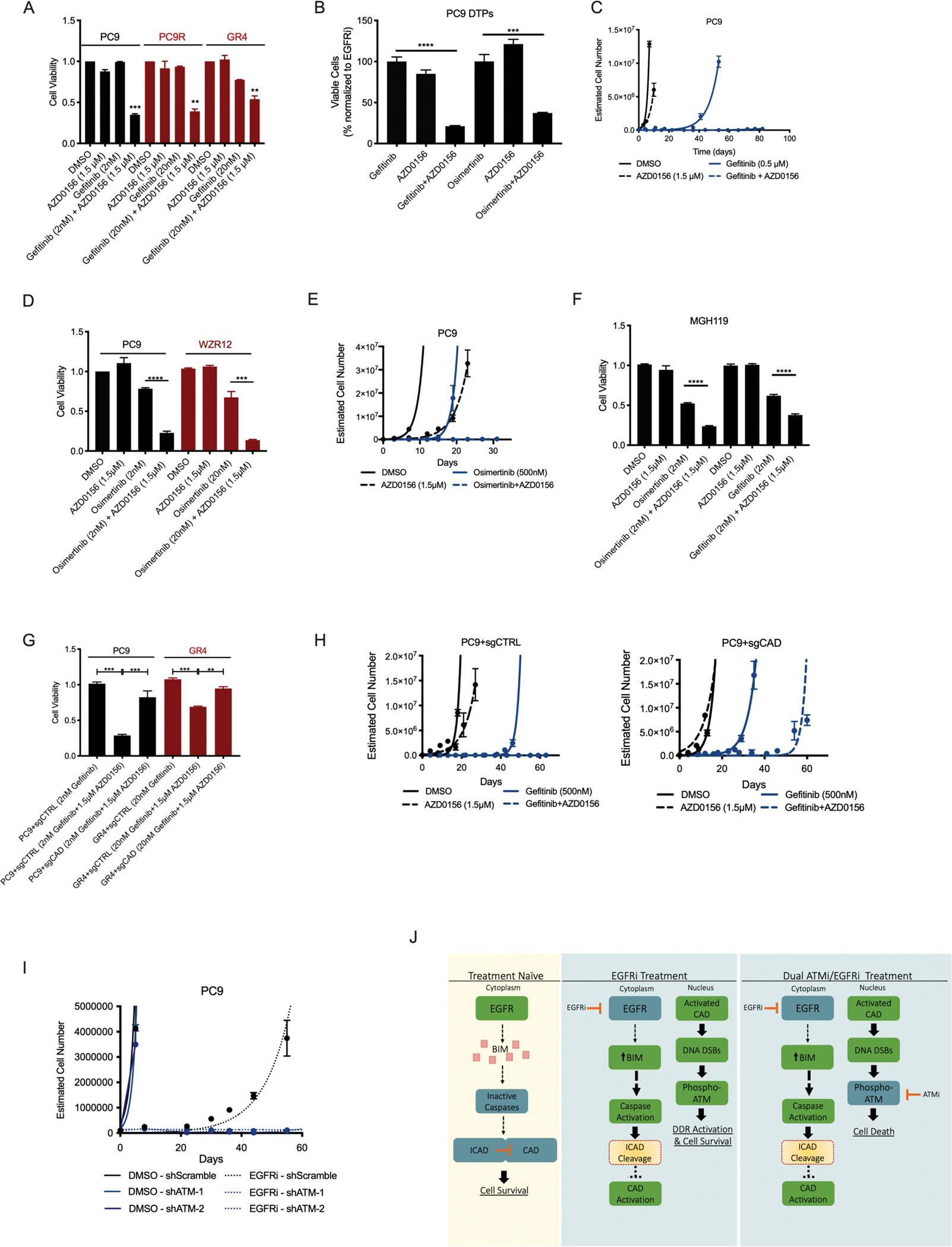
Effect of ATM inhibition on survival and growth of EGFR inhibitor-resistant cells. **A**. Cell viability in the indicated drug treatment conditions in EGFR inhibitor-sensitive (PC9) and -resistant (PC9R, GR4) cells. **B**. Cell viability, as assessed through the percentage of surviving cells (normalized to gefitinib-only treated), of PC9 drug-tolerant persisters (DTPs) following 4-day treatment with single-agent gefitinib (100 nM), AZD0156 (1.5 μM), or combination. **C**. Estimated cell number during long-term time-to-progression (TTP) assay of PC9 cells treated with gefitinib, AZD0156 or the combination. **D**. Cell viability in the indicated drug-treatment conditions in EGFR inhibitor-sensitive (PC9) and -resistant (WZR12) cells. **E**. Estimated cell number during long-term time-to-progression (TTP) assay of PC9 cells treated with osimertinib, AZD0156, or the combination. **F**. Cell viability in the indicated drug treatment conditions in EGFR inhibitor-sensitive MGH119 cells. **G**. Cell viability in the indicated drug treatment conditions in EGFR inhibitor-sensitive (PC9) and -resistant (GR4) cells with or without CAD presence (sgCTRL or sgCAD, respectively). **H**. Estimated cell number during long-term time-to-progression (TTP) assay of PC9 cells treated with gefitinib, AZD0156, or the combination, with or without CAD presence (sgCTRL or sgCAD, respectively). **I.** Estimated cell number during long-term time-to-progression (TTP) assay of PC9 cells treated with vehicle or gefitinib, with or without ATM presence (shScramble or shATM, respectively). **J.** Conceptual diagram linking EGFR inhibition to ATM activation and dependence. N=3 for all cell viability assays and estimated cell number during long-term time-to-progression assays presented, where the mean ± S.E.M is plotted. *P* values were determined using unpaired, two-tailed Student’s *t*-tests. ** refers to P<0.01, *** refers to P<0.001, and **** refers to P<0.0001.

The results above suggest that cells surviving treatment with targeted therapies may require ATM activity in order to resolve DSBs caused by targeted therapy exposure. This idea implies that ATM inhibition may have therapeutic value as a means of improving the depth and duration of responses to targeted therapies. To explore this hypothesis, we first treated cells with AZD0156, which blocked geftinib-induced ATM activation (Fig. 2B). ATM inhibition sensitized EGFR inhibitor-sensitive and -resistant cells to gefitinib, despite the fact that ATM inhibition was not associated with single agent toxicity at these concentrations (Fig. 3A and fig. S3A). Additionally, we confirmed that pharmacological inhibition of ATM conferred synergistic sensitization to EGFR inhibition in PC9 cells (combination index (CI) < 1.0 by Chou-Talalay method, Table S1; (18)). This effect was associated with combination therapy-induced increases in caspase 3 cleavage and Annexin-V staining (fig. S3B and S3C), suggesting that increases in DSB formation caused by combined oncogene targeted therapy and ATM inhibition results in cell death by apoptosis.

The notion that cells surviving EGFR inhibitor therapy are nevertheless sensitive to combined EGFR and ATM inhibition suggests that this combination strategy may be an effective means of ablating cells that survive upfront treatment with EGFR inhibitor monotherapy. To directly test this concept, PC9 cells were treated with an EGFR inhibitor dose 100-fold greater than the IC_50_ of these cells (2μM gefitinib or 1μM osimertinib) for 9 days, selecting for a population of cells which are termed drug tolerant persisters (DTPs) (19). Reflecting their exposure to EGFRi, DTP cells had higher expression of p-ATM compared to untreated, gefitinib-sensitive PC9 cells (fig. S3D). Consistent with our mechanistic studies, we observed the presence of cytochrome c in the cytoplasm of DTP cells, implying MOMP and caspase activation in these cells (fig. S3E). Further, ATM inhibition sensitized these cells to EGFR blockade with gefitinib or osimertinib (Fig. 3B). Because resistance eventually emerges from cells that survive upfront drug treatments, we next hypothesized that combined EGFR and ATM inhibition may delay resistance evolution. Indeed, in a long-term qualitative time to progression (TTP) assay (20), wherein cell population size is monitored over weeks during drug treatment to model the development of resistance in vitro, we observed that AZD0156 had only a minor effect on cell growth, and gefitinib monotherapy led to resistance outgrowth in around 40 days, whereas treatment with combined gefitinib and AZD0156 treatment effectively eradicated residual cells, leading to long-term suppression of resistance outgrowth (Fig. 3C). Similar results were seen following the use of the third-generation EGFR inhibitor, osimertinib, in EGFR inhibitor-sensitive and -resistant populations of cells, both in short-term (three day) experiments (Fig. 3D) as well as in a long-term (32 day) TTP assay (Fig. 3E). These long-term TTP findings were recapitulated in HCC827 cells (fig. S3F), and in two *KRAS* mutant cell lines treated with the ERK inhibitor SCH772984, expanding the application of the ATM inhibitor plus targeted therapy concept to non-EGFR driven cell line models (fig. S3F–G). Similar results were also observed in MGH119 cells, which were recently derived from a treatment-naïve, *EGFR* mutant NSCLC patient (21, 22), with respect to EGFR inhibitor-induced γ-H2AX formation, ATM activation, and ATM inhibitor-mediated sensitization to EGFR blockade (Fig. 3F and fig. S3H). Additionally, MGH119 drug-tolerant persisters (DTPs) showed a similar sensitization to the EGFR inhibitor gefitinib when treated in combination with AZD0156 (fig. S3I). ATM inhibitor-mediated sensitization to targeted therapy was only observed in cells treated with their cognate targeted therapy, as evidenced in a panel of *KRAS* mutant cell lines treated with gefitinib, to which they are relatively insensitive, or the ERK inhibitor SCH772984, to which they are sensitive (fig. S3J–K). Finally, having determined that DSB formation and ATM activation occur in a CAD-dependent manner following EGFR blockade, we assessed the impact of CAD on the cellular response to combined EGFR and ATM inhibition. CRISPR/Cas9-mediated CAD knockout rescued the toxicity of the dual therapy in short-term assays conducted in both EGFR inhibitor-sensitive and –resistant cells (Fig. 3G). Similarly, in long-term TTP assays in PC9 cells, we observed that CAD knockout (sgCAD) led to the outgrowth of cells in the context of dual EGFR plus ATM inhibition, whereas cells expressing CAD (sgCTRL) were durably growth suppressed as expected (Fig. 3H). Lastly, we used RNAi technology to knockdown expression of ATM and found that loss of ATM abrogated gefitinib-induced γ-H2AX induction and phenocopied the ATM inhibitor AZD0156 in long-term TTP assays in PC9 cells, suggesting that AZD0156 functions in these contexts through on-target ATM inhibition (Fig. 3I and fig. S3L). Together, these results demonstrate that CAD-mediated formation of DSBs in cells surviving treatment with EGFR inhibitors imposes a synthetic dependence on ATM, a kinase critical for the resolution of this DNA damage. Thus, combined EGFR and ATM inhibition eradicates cell populations that otherwise survive EGFR inhibitor monotherapy, leading to long-term, CAD-dependent suppression of resistance outgrowth (Fig. 3J).

### Pharmacological targeting of PARP sensitizes cells to EGFR inhibition

Having established that ATM inhibition can be used to effectively target cancer cells that survive EGFR inhibitor therapy, we sought to evaluate whether these findings could be extended to other DDR pathway inhibitors through a similar mechanism. Specifically, we focused on the PARP inhibitor olaparib, which is FDA-approved and indicated for use in multiple cancer contexts. After performing western blots showing the effects of single agent EGFRi (gefitinib) and PARPi (olaparib) treatment on PC9 cells (fig. S3M), we performed a similar panel of experiments to assess the effect of this combination on cell viability. The combination of EGFR plus PARP inhibition led to qualitatively similar but more modest effects than combined EGFR plus ATM inhibition in short term assays performed in both parental PC9 cells and resistant derivatives treated with first or third generation EGFR inhibitors (fig. S3N–O). In long-term TTP assays in PC9 cells, this combination mirrored more closely the effect of the EGFR plus ATM inhibitor combination (fig. S3P). Lastly, consistent with findings in combined EGFR plus ATM inhibitor-treated cells, CAD knockout rescued the viability of both EGFR inhibitor-sensitive and -resistant cells treated with the combination of EGFR and PARP inhibitors (fig. S3Q). Thus, CAD-driven DNA damage in cells surviving treatment with EGFR inhibitors also creates a synthetic dependence on PARP.

### Combined inhibition of ATM and EGFR forestalls the outgrowth of tumors in vivo

**Figure 4:**
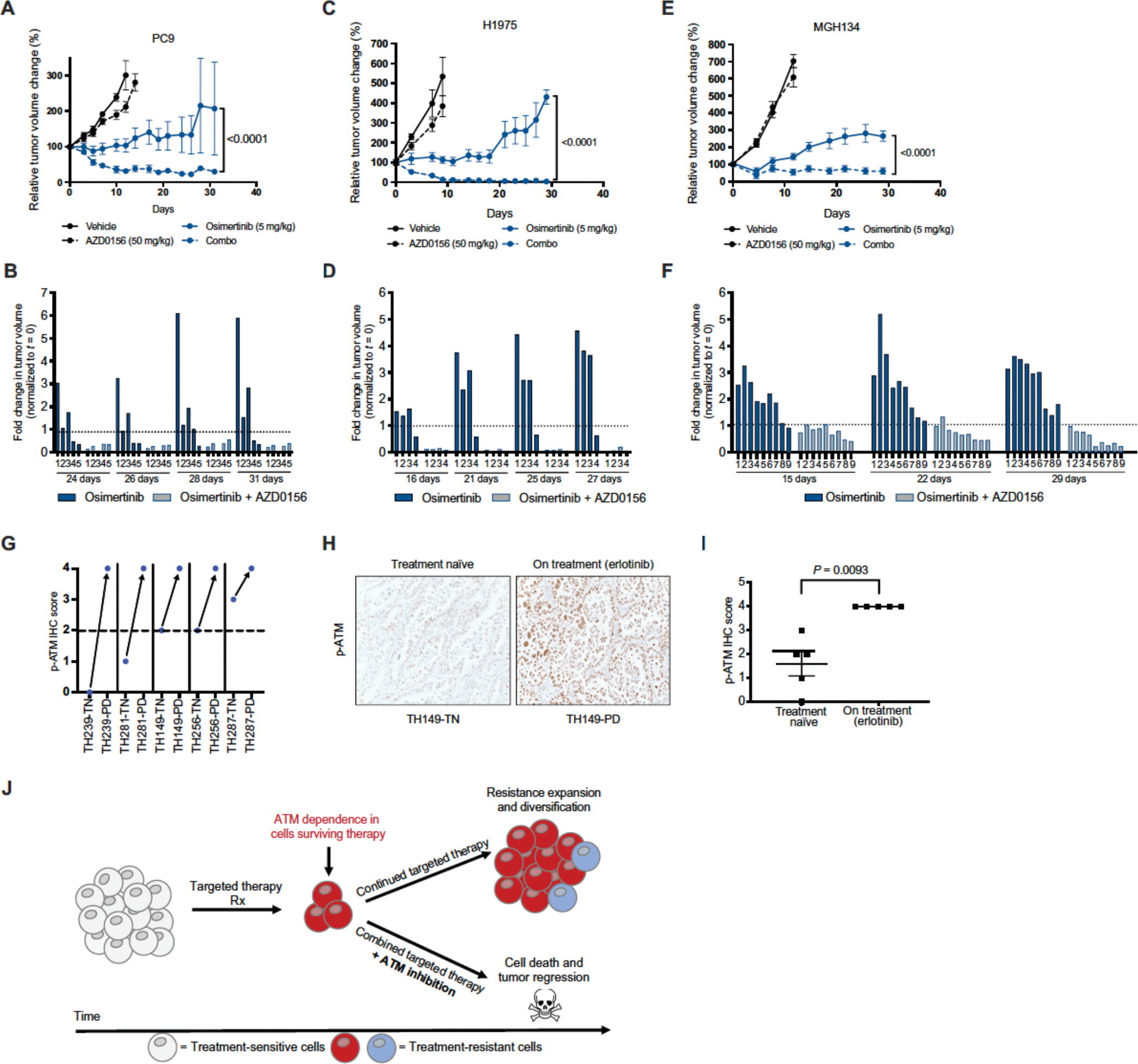
Targeted therapy-induced ATM activation and targeting in vivo. **A**. Tumor volume (normalized to t=0, %) of PC9 cell line xenografts in nude mice following treatment with vehicle, osimertinib, AZD0156, or the combination for indicated time points (*n*=5 mice in each treatment arm). *P* values were determined using unpaired, two-tailed Student’s *t*-test. **B**. Fold change in individual tumor volume (normalized to t=0) for PC9 tumors treated with osimertinib or the combination of osimertinib and AZD0156. **C**. Tumor volume (normalized to t=0, %) of H1975 cell line xenografts in nude mice following treatment with vehicle, osimertinib, AZD0156, or the combination for indicated time points (*n*=4–5 mice in each treatment arm). *P* values were determined using unpaired, two-tailed Student’s *t*-tests. **D**. Fold change in individual tumor volume (normalized to t=0) for H1975 tumors treated with osimertinib or the combination of osimertinib and AZD0156. **E**. Tumor volume (normalized to t=0, %) of MGH134 patient-derived cell line xenografts in nude mice following treatment with vehicle, osimertinib, AZD0156, or the combination for indicated time points (*n*=9–10 mice in each treatment arm). *P* values were determined using unpaired, two-tailed Student’s *t*-tests. **F**. Fold change in individual tumor volume (normalized to t=0) for MGH134 tumors treated with osimertinib or the combination of osimertinib and AZD0156. **G**. p-ATM IHC score of patient tumor tissue obtained before (treatment naïve, TN) or during treatment with erlotinib (progressive disease, PD). Same numbers indicate tumors longitudinally sampled from the same patient. **H**. Representative image of p-ATM IHC from patient tumors before treatment (treatment naïve) or during treatment (progressive disease). Images taken at 20x magnification. **I**. p-ATM IHC scores from 5 matched tumor samples from patients with *EGFR* mutant lung adenocarcinoma taken at the time of diagnosis and at the time of relapse to EGFR inhibitor erlotinib. *P* values were determined using unpaired, two-tailed Student’s *t*-tests. **J**. Proposed model of ATM dependence in targeted therapy treated tumors, leading to rational combination of targeted therapies and ATM inhibitors.

To test the efficacy of the EGFR plus ATM inhibitor drug combination in vivo, a xenograft study was performed. Once subcutaneous PC9 tumors reached 100–200 mm^3^, mice were randomized into one of four treatment arms including vehicle, single agent EGFR inhibitor osimertinib, single agent ATM inhibitor AZD0156, or the combination treatment. Whereas ATM inhibition alone had little effect on tumor growth, EGFR inhibitor monotherapy suppressed the growth of tumors before eventually giving rise to resistance outgrowth (Fig. 4A–4B). In contrast, mice treated with the combination of ATM and EGFR inhibitors displayed sustained tumor regressions that lasted throughout the length of the study. We observed no apparent toxicity based upon body weight of the mice in this study (fig. S4A). On-target activity of the ATM and EGFR inhibitors, and osimertinib-induced ATM activation, were each verified via immunoblotting of mouse tumor lysates following treatment (fig. S4B–C). These findings were confirmed in an additional *EGFR* mutant H1975 lung cancer xenograft model (Fig. 4C–D and fig. S4D–E). Finally, we used a panel of three cellular models recently derived from patients with lung cancer, which included MGH134 from a patient with *EGFR* mutant NSCLC who developed resistance to first line erlotinib therapy via a *EGFR*^*T790M*^ resistance mutation, MGH1109 from a treatment naïve patient with *EGFR* mutant NSCLC, and MGH006 from a treatment naïve patient with *EML4*-*ALK* variant 1 mutant NSCLC. We observed that ATM inhibition suppressed the outgrowth of resistance to matched targeted therapies in long-term TTP assays (fig. S4F). Consistent with this finding, osimertinib treatment yielded initial growth suppression followed by eventual tumor progression in MGH134 xenograft-bearing mice, whereas the combination of osimertinib plus AZD0156 yielded sustained tumor regressions that lasted throughout the length of the study (Fig. 4E–F).

### ATM activation is observed in tumors from patients treated with EGFR inhibitors

Finally, to investigate potential clinical correlates of these findings, we first performed immunohistochemical (IHC) staining to quantify p-ATM S1981 expression in matched tumor samples taken from patients with *EGFR* mutant NSCLC before (treatment naïve, TN) and during (progressive disease, PD) treatment with the EGFR inhibitor erlotinib. In 5 of 5 cases, p-ATM staining was increased in tumors progressing on treatment with erlotinib relative to matched pre-treatment tumor samples (Fig. 4, G and H). Aggregate analysis of all patient samples correspondingly revealed a significant increase in p-ATM expression in progressing tumors undergoing erlotinib treatment (Mann-Whitney test, *P*=0.0079, Fig. 4I). Next, we hypothesized that rare patients with *EGFR* mutant NSCLC whose tumors harbor co-occurring loss-of-function mutations in *ATM* (<5% of patients) may exhibit more durable responses to EGFR kinase inhibitors than those whose *EGFR* mutant tumors lack loss-of-function *ATM* mutations. We queried the Memorial Sloan Kettering integrated mutation profiling of actionable cancer targets (MSK-IMPACT) Clinical Sequencing Cohort database, which contained data on the time to clinical progression on first-line erlotinib therapy for 11 patients whose tumors contained co-occurring *EGFR* activating/erlotinib sensitizing mutations and *ATM* mutations (Table S2). We annotated these *ATM* mutations as being either likely loss-of-function, nonsense mutations and those annotated as deleterious/damaging using the SIFT and PolyPhen-2 tools, or likely non-functional. Time to progression on erlotinib in patients whose tumors harbor co-occurring loss-of-function *ATM* mutations was 17.8 +/− 10.9 months compared to 9.0 +/− 1.9 months in those patients whose tumors harbor likely non-functional *ATM* mutations (*P* < 0.05) (fig. S4G). Although the results of this analysis should be considered with care given the small sample size, we note that the latter figure is consistent with the time to clinical progression of 8–12 months observed in multiple studies of unselected patients with *EGFR* mutant NSCLC treated with first-line erlotinib therapy, including one study which used the same clinical cohort at the same institution (23). Together, these data suggest that ATM activation occurs in human tumors surviving treatment with EGFR inhibitors, where it likely plays a tumor protective role. A summary of the key results of this study are summarized in the schematic shown in Fig. 4J.

## Discussion

The heterogeneous and multi-focal nature of acquired resistance mechanisms to targeted therapies limits our ability to effectively treat and reverse resistance after it emerges (4–7). As a consequence, substantial efforts are now being expended to develop upfront treatment strategies capable of forestalling resistance evolution. To date, these strategies include upfront targeting of prevalent resistance mechanisms or points of convergent signaling downstream of key resistance mechanisms, identifying and targeting vulnerabilities unique to the drug tolerant persister cells that survive initial treatment with oncogene targeted therapies, and identifying and targeting “collateral sensitivities,” which are scenarios where acquired resistance to an initial therapy produces heightened sensitivity to a second therapy (4–7, 24–30). Here, we demonstrate that by targeting ATM-dependent survival in cells undergoing treatment with targeted therapies, it is possible to increase the depth and duration of activity of those therapies. Importantly, because DNA damage induction may occur in both cells with pre-existing resistance mechanisms and in drug-tolerant persister cells, we speculate that this approach may have advantages over strategies targeting only one of these two sources of resistance.

This work also fits into the broader context of targeting the DDR pathway as a cancer therapeutic strategy. The discovery that PARP inhibitors have specific activity in the context of *BRCA1/2* mutations led to the subsequent, successful deployment of PARP inhibitors for the treatment of certain *BRCA* mutant and HR-deficient tumors (31). This advance helped catalyze the development of additional agents targeting key nodes in the DDR network, including ATM, as well as the search for mutational contexts in which these drugs exhibit activity and mechanism-based combination therapies to enhance their activity (31). To date, however, many tumors lack mutations that confer sensitivity to these agents. Further, most clinically advanced combination therapies involving these agents involve the use of DNA damaging chemotherapies, inhibitors of other DDR pathway nodes, or modifiers of chromatin state, strategies which may cause increased toxicity to both tumor and normal cells (31). Clinical trials involving combinations of PARP inhibitors with chemotherapies like temozolomide, cisplatin, and gemcitabine have revealed exacerbated toxicity that required dose reductions, implying a narrow therapeutic index (31). Our present demonstration that oncogene targeted therapies potentiate ATM inhibitor action through caspase activation, which has been shown to be specific to cells harboring sensitizing oncogenic driver mutations, is thus particularly promising because it may enable tumor-selective activation of lethal DNA damage.

Several open questions and potential limitations should be considered. First, although caspase activation is believed to be a common feature of tumor cells treated with oncogene-matched targeted therapies, and although we demonstrate targeted therapy-induced ATM activation in diverse oncogene-driven models, the full breadth of scenarios in which ATM inhibition may be used to potentiate the activity of targeted therapies is yet to be determined. Second, although our studies suggest that TP53 mutational status does not influence cellular responses to combined EGFR and ATM inhibition (Table S3), it remains to be determined whether other recurrent mutations, for example in DDR pathway genes, influence responsiveness to these combination therapies. Finally, a growing body of work suggests that DNA damage induction by PARP inhibitors may potentiate not only the toxicity of DNA damaging chemo- and radiation therapies, but also immune surveillance and checkpoint blockade. Thus, a key question for future studies is whether DNA damage secondary to combined targeted therapy plus ATM inhibition can potentiate inflammatory signaling, immune surveillance, and checkpoint inhibitor activity in tumors.

Together, the demonstration that ATM inhibition potentiates tumor responses to oncogene targeted therapies is well positioned for near term clinical development. Multiple selective ATM kinase inhibitors are currently in clinical development, including those with blood-brain barrier permeability (32), and preclinical studies suggest that these agents may have very favorable toxicity profiles. Thus, the studies presented here provide a clear, mechanism-based rationale for the near-term design of clinical trials in diverse malignancies currently treated with standard-of-care targeted therapy paradigms.

## Materials and Methods

### Study Design

The overall goal of this study was to determine the mechanism and translational implications of oncogene targeted therapy-induced DNA damage. Specifically, this study focused primarily on EGFR inhibitor targeted therapies in the context of *EGFR* mutant NSCLC and whether selective ATM inhibition could improve the depth and duration of response to these agents.

In vivo studies were performed using 6- to 8-week-old female nude mice. The number of mice used in each experimental group was determined on the basis of statistical power analysis to render statistical significance of the experimental data between different experimental groups and ranged from five to six mice per group. Before treatment, mice were randomized on the basis of tumor volume to ensure evenly distributed average tumor sizes across each group. Mouse survival end points were based on the maximum tumor volume allowed under the approved animal use protocol (1000 mm^3^). Investigators received measurements of tumors with each treatment group, and so were blinded from the actual treatment of mice in each group. Tumors that did not take (no viable tumors formed) were excluded from the study. All mouse studies were performed under protocols approved by the Institutional Animal Care and Use Committee and the institutional review board of Duke University School of Medicine. The mice were housed in an animal facility that is free of specific pathogens. All mice were fed standard normal chow diet and housed under controlled temperature and 12-hour light/12-hour dark cycle conditions. The mice were under the general supervision of experienced veterinarians and were attended and monitored at least daily by a trained animal care technician.

### Cell lines and reagents

All cell lines were maintained in a humidified incubator at 37 °C with 5% CO2. PC9, HCC827, H3122, A549, A375, GR4, WZR12, PC9R, MGH134, MGH006, MGH1109, and MOLM13 were cultured in RPMI 1640 medium with 10% fetal bovine serum (FBS) and 1% penicillin–streptomycin. MIA PaCa-2 and SW1573 were cultured in DMEM/F-12 medium with 10% fetal bovine serum (FBS) and 1% penicillin–streptomycin. MGH119 were cultured in DMEM medium with 10% fetal bovine serum (FBS) and 1% penicillin–streptomycin. 293FT cells were cultured in DMEM high glucose medium with 10% FBS, 1% penicillin–streptomycin, 1% sodium pyruvate, 1% nonessential amino acids and 1% GlutaMax. All cell lines were purchased from American Type Culture Collection or Duke University Cell Culture Facility except for MGH lines, which were obtained from Dr. Aaron Hata. Cell lines were authenticated using the Promega PowerPlex 18D kit. Drugs were purchased from ApexBio (Osimertinib, Gefitinib, SCH772984, Ceritinib, Quizartinib, PLX4720, AMG510, Lorlatinib), Cayman Chemical (Olaparib), and SelleckChem (AZD0156, AZD1390, Q-VD-Oph, Cycloheximide).

### Evolving drug resistant cell lines and drug-tolerant persisters (DTPs)

To achieve drug resistance in vitro, PC9 cells were continuously cultured in increasing concentrations of drugs. Cells were first drugged at a dose approximately equal to their GI50 value (concentration for 50% of maximal inhibition of cell proliferation). The growth rate was monitored with weekly passaging and the concentration of drug was increased once a stable growth rate was achieved. Drug tolerant persister (DTP) cells were derived by treating drug-sensitive PC9 and MGH119 cells with the relevant drugs at concentrations greater than 100 times the established IC50 values (2 μM for gefitinib, 1 μM for osimertinib), for three successive rounds of culture, with each treatment lasting 72h. Viable cells remaining attached on the dish at the end of the third round of drug treatment were considered to be DTPs and were collected for use in subsequent analyses.

### Short-term cell viability assays

For GI_50_ dose-response assays, cells were seeded into 96-well plates at a density of 4,000 cells per well. 24h following plating, cells were treated with vehicle (DMSO) or a ten-fold serial dilution of drug. Each treatment condition was conducted in triplicate. Three days following the addition of drug, cell viability was quantified using Cell Titer Glo (CTG, Promega). The relative cell viability was determined by normalizing the raw luminescence values for each treatment condition to DMSO-treated wells. For experiments involving two drugs, slight modifications were made. One drug was kept at a constant concentration across all wells and a serial dilution of a second drug was added on top of the background drug. One set of triplicate wells was treated with DMSO only and one set of triplicate wells was treated with background drug only. The relative cell viability was normalized to the luminescence of the background drug only. Dose–response curves were fit using GraphPad/Prism 7/8 software. Cell viabilities were reported as the percentage of cells (relative to DMSO-treated cells) that survived treatment at the indicated dose(s) of drug.

For bulk growth assays, cells were seeded in 10-cm plates at the following densities: PC9– 1×10^6^, HCC827– 150,000, MOLM13–1×10^6^, PC9R-500,000, GR4–500,000, WZR12–500,000 cells. Twenty-four hours following plating, cells were treated with vehicle (DMSO) or the indicated dose of targeted therapy for 24h. Each treatment was conducted in triplicate. Following 24h treatment, cells were harvested to obtain raw cell counts, reported as cell number. For DTPs, PC9 and MGH119 cells that remained following 9-day treatment with gefitinib or osimertinib were harvested and re-plated. DTP cells were treated with the appropriate EGFR inhibitor, AZD0156, or the combination of the two drugs for 4 days. Each treatment was conducted in triplicate. The percentage of viable cells (% of cells surviving treatment, normalized to EGFR inhibitor-only treated cells) is reported.

### Annexin V Staining

Annexin V staining was performed to determine the percentage of cells undergoing apoptosis. 100,000 cells were plated in six-well plates and 24h later were treated with the indicated doses of vehicle (DMSO), targeted therapy, AZD0156, or the combination of drugs for 24h. Upon collection, cells were washed twice with PBS, resuspended in 100uL 1X Annexin V binding buffer (BD Biosciences) containing 5uL Annexin V stain conjugated to APC (allophycocyanin) (BD Biosciences) and 5uL 7-AAD (BD Biosciences). Phosphatidylserine externalization was measured using APC-conjugated Annexin V and 7-AAD was used as a viability probe. Following a 15min incubation at RT, the samples were analyzed using the flow cytometer BD FACSCanto II. The gating strategy was defined using untreated/unstained cells. Analysis of flow cytometry data was performed with FlowJo v10.

### Cloning of Constructs

CRISPR constructs were cloned following previously published methods (33) using previously characterized sgRNAs (34). sgRNA inserts were synthesized by CustomArray of the form:

GGAAAGGACGAAACACCGxrefxrefxrefXXGTTTTAGAGCTAGAAATAGCAAGTTAAAATAAGGC “X” denotes unique 20mer sgRNA sequence (see “20-mer sequences” below). The oligo pool was diluted 1:100 in water and amplified using NEB Phusion Hotstart Flex enzyme master mix and the primers ArrayF and Array R.

ArrayF: TAACTTGAAAGTATTTCGATTTCTTGGCTTTATATATCTTGTGGAAAGGACGAAACACCG

ArrayR: ACTTTTTCAAGTTGATAACGGACTAGCCTTATTTTAACTTGCTATTTCTAGCTCTAAAAC

PCR protocol: 98°C/30 s, 18 × [98°C/10 s, 63°C/10 s, 72°C/15 s], 72°C/3 min. Inserts were cleaned with Axygen PCR clean-up beads (1.8x; Fisher Scientific) and resuspended in molecular biology grade water. LentiCRISPRv2 (Addgene ID 52961) was digested with BsmBI (Thermo Fisher) for 2 h at 37°C. The ~13 kB band was gel-extracted after size-selection on a 1% agarose gel. Using 100 ng of cut lentiCRISPRv2 and 40 ng of sgRNA oligos, a 20 mL Gibson assembly reaction was performed (30 min, 50°C). Following Gibson assembly, 1 mL of the reaction was transformed into electrocompetent Lucigen cells, spread on LB-ampicillin plates and incubated overnight. Single colonies were picked and grown overnight in liquid culture at 37°C. Plasmid extraction was performed using a Plasmid miniprep kit (Qiagen). DNA was used to make lentivirus as described below. shRNA glycerol stocks were obtained from the Duke Functional Genomics Core Facility. Glycerol stocks were streaked out on LB/Amp plates overnight. Subsequently, colonies were picked and grown overnight in liquid culture at 37°C. Plasmid extraction was performed using a Plasmid miniprep kit (Qiagen). DNA was used to make lentivirus as described below.

### Lentivirus Production and Transduction

HEK 293 T cells were grown in 10 cm to ~50% confluence. Per-plate transfection was performed using Fugene6 (Promega), 6.2 mg of psPAX2, 0.620 mg pVSVg, 6.25 mg of CRISPR plasmid. After 30 min of incubation at room temperature, the mixture was added to the cells and incubated overnight. The next day harvest media was added (DMEM 30% FBS). After two consecutive 24h collections, the harvested virus was passed through a 0.45 μm filter. Transductions were performed by plating 200,000 cells in 2mL RPMI media into six-well dishes. The following day, 0.5mL virus and 2 μg polybrene were added to each well of the 6-well plate. The cells were then centrifuged at 2500 RPM for 1 hour at 37°C and incubated overnight at 37°C. 24h later, cells were selected with puromycin (2ug/mL).

### Immunoblotting

Immunoblotting was performed as previously described (35), with slight modification. Protein lysates were prepared with RIPA lysis buffer supplemented with 1× protease inhibitor cocktail. Crude lysates were cleared using QIAshredder Homogenizers (Qiagen) and centrifuged at 13,000 r.p.m. for 2 min at 4 °C. Membranes were probed with the following primary antibodies: β-actin (Cell Signaling Technology (CST) no. 4970), p-ATM (S1981) (Abcam, ab81292), ATM (CST no. 2873), γ-H2AX (p-Histone H2A.X) (CST no. 9718), Vinculin (CST no. 4650), ATR (CST no. 2790), pChk2 (T68) (CST no. 2661), p-Chk1 (S317) (CST no. 2344), Caspase 9 (CST no. 9502), Caspase 3 (CST no. 9662), Cleaved Caspase 3 (CST no. 9661), Caspase 7 (CST no. 9492), BIM (CST no. 2933), BAK (CST no. 3814), BAX (CST no. 2772), ICAD (Santa Cruz, sc17818), CAD (Santa Cruz, sc374067), p-EGFR (CST no. 2234), T-EGFR (CST no. 4267), p-ERK (CST no. 4370), T-ERK (CST no. 4695), p-MEK (CST no. 9127), p-FLT3 (CST no. 4577), EXO1 (Abcam, ab95068), BRCA1 (Santa Cruz, sc6954), BRCA2 (Santa Cruz, sc8326), RAD51B (Santa Cruz, sc377192), DNA polymerase iota (Abcam, ab157244), p-AKT S473 (CST no. 9271), T-AKT (CST no. 9272), Chk1 (CST no. 2360), Chk2 (CST no. 6334), H2AX (CST no. 7631), Cytochrome c (CST no. 11940). Primary antibodies were diluted 1:1,000 in 5% BSA and incubated overnight (16 hours). Following incubation with HRP-conjugated secondary antibody, blots were developed with SuperSignal West Pico PLUS Chemiluminescent Substrate (ThermoFisher) or ECL Western Blotting Substrate (ThermoFisher). For cell fractionation experiments, the cell fractionation kit (CST #9038) was used according to the manufacturer’s instructions. The Bradford method was additionally used to normalize protein concentrations of all samples in these experiments, including the cytoplasmic and membrane-bound fractions.

### Time-to-Progression Assay

To evaluate the relative ability of treatments to delay the reemergence of logarithmic cell growth in vitro (resistance), cells were plated in triplicate in 6 cm plates at 100,000 cells per plate in normal growth media. After 24h, the growth media was replaced with the indicated treatment. At the time points indicated, the cells were lifted with 0.25% trypsin (Life Technologies) and counted using a Z2 Coulter Particle Count and Size Analyzer (Beckman Coulter, Pasadena, CA). All cells up to 100,000 were centrifuged at 1200 RPM for 5 minutes and resuspended in 3 mL of media and then plated in a 6 cm plate with fresh treatment. This procedure was repeated weekly for 4–12 weeks, depending on the kinetics of resistance. Weekly growth rates (μ) were calculated from the number of cells plated the previous week (N0) and the number counted the current week (N) according to the formula ln N = ln N_0_ + μ*t; where t is elapsed time. These growth rates were then used to project the total virtual cell number.

### Neutral Comet Assay

Trevigen Kit was used according to the neutral comet assay protocol conditions (Trevigen, 4250–050-K). DMSO (vehicle) was used as a negative control, and 10 Gy irradiation immediately prior to harvest was used as a positive control. All vehicle and drug treatments were performed for 24h. Cells were imaged on the Live Cell Station 1: Zeiss Axio Observer inverted microscope using the following specifications: 10X magnification on the GFP (488nm) channel, 450ms exposure time. Comet analysis was done using CellProfiler. Pipeline was optimized using negative control and positive control images, only. Comets with no comet head (debris) were thrown out. For quantification, extent tail moment was calculated as follows: Extent Tail Moment = Tail DNA% × Length of Tail. Results show are the mean extent tail moment and the standard error of the mean (SEM) obtained from several hundred images per treatment condition.

### Immunofluorescence

Immunofluorescence assay to detect Rad51 foci was performed as previously described(36, 37), with minor modifications. Cell lines were plated on glass coverslips and the following day were treated with 100 nM gefitinib, 1.5 μM AZD0156 and/or Q-VD-OPh for 24h. Cells were fixed with 3% paraformaldehyde (20 min at room temperature). The cells were then washed 4 × 15 min in PBS-T (PBS containing 0.15% BSA and 0.1% Triton-X-100). Slides were then incubated with 200 ng/ml anti-Rad51 (Santa-Cruz) overnight, washed in PBS-T and incubated with Alexa-488 conjugated goat anti-rabbit IgG (CST) at 1:1000 dilution for 1 hour. Finally, the cells were washed three times with PBS-T and mounted using Prolong Gold anti-fade reagent with DAPI (Life Technologies). The slides were then imaged using a Leica SP5 inverted confocal microscope with ×40 oil objective. For all representative images in the manuscript, experiments were conducted at least twice, and had no repeatability issues. Percentage of Rad51+ cells was calculated by visual scoring of cells in the images obtained, with only cells having 5 or greater GFP-staining foci being termed Rad51+. 50–100 cells were scored/treatment condition.

### Immunohistochemistry (IHC)

All patient tumor samples analyzed were obtained under institutional review board–approved protocols with informed consent obtained from each patient under the guidance of the University of California, San Francisco (UCSF). All relevant ethical regulations were followed. The mutational status of EGFR or other known drivers or resistance was determined using FoundationOne (Foundation Medicine), or internal UCSF ​molecular pathology evaluation (UCSF 500). Tissues were fixed in 10% formalin overnight and embedded in paraffin. Tissue sections of PDX and patient samples were sectioned on slides with 4-μm thickness. Following IHC preparation of slides and staining via p-ATM antibody, slides were imaged and analyzed for staining intensity. Images were taken at 20x magnification using the Olympus BX46 light microscope. The analysis was based on the staining intensity and percentage of cells staining positive for p-ATM. The staining area was scored using the following scale, 0: 0–10%, 1: 10–20% of tissue stained positive, 2: 20–40% stained positive, 3: 40–70% stained positive and 4: > 70% positive cells. Average IHC scores were generated from visualization of 3 different areas of the slide for each sample.

### In vivo studies

All animal procedures and studies were approved by the Institutional Animal Care and Use Committee (IACUC) at Duke University. NSCLC cell lines (PC9, H1975, or MGH134) were evaluated by IMPACT testing prior to their use in vivo. ~0.5–1 × 10^6 cells were suspended in a PBS and Matrigel solution (PBS: Matrigel = 1:1), and 100μl of cell suspension was subcutaneously injected into the flank of ~6–8 week-old female nude mice. Tumor size was measured three times weekly with calipers and tumor volume was calculated by the formula, V = L × W^2^ × 0.52 (L = longest diameter, W = shortest diameter). When tumor volume reached ~100–200 mm^3^, mice were randomized into treatment groups, with each group having 5–6 mice. AZD0156 and osimertinib were purchased from SelleckChem (US). AZD0156 was resuspended in Ora-Plus suspension (clinical grade, purchased from Duke Pharmacy Stock Room), and osimertinib was dissolved in a 10% DMSO, 30% PEG400 and 60% H_2_O solution. All drugs were administered orally with 100μl drug suspension/dose per mouse. AZD0156 was administrated at 50mg/kg daily, and osimertinib was administrated at 5mg/kg daily. All mice were dosed Monday-Friday (5 days/week). Tumor size was monitored two to three times per week until the end point when tumors reached ~1,000 mm^3^ or tumors were ulcerated.

### Statistical analyses

All results are shown as mean ± s.e.m, unless otherwise shown. *P* values were determined using unpaired, two-tailed Student’s *t*-tests, Mann-Whitney test, or, for grouped analyses, one-way ANOVA with Tukey’s post hoc test, and *P* < 0.05 was considered significant. Unless otherwise noted, all experiments were performed a minimum of three times and measurements were taken from distinct biological replicate samples.

## Supplementary Material

Figure data (tabular format)

supplemental material

## Data Availability

All data associated with this study are present in the paper or supplementary materials.
