# Supplementary material for: Small-molecule targeted therapies induce dependence on DNA double-strand break repair in residual tumor cells
Source: Sci Transl Med. Author manuscript; Available in PMC 2022 Sep 28. (PMC9516479; doi:10.1126/scitranslmed.abc7480)
Supplement: supplemental material [file NIHMS1836154-supplement-supplemental_material.docx]

**Supplementary Figures**

**Figure S1: Additional characterization of sub-lethal dose treatment of cancer cells. A.** Immunoblot of PC9, HCC827, H3122, A549, A375, MOLM13 and MIA PaCa-2 cells treated with doses of targeted therapies in Figure 1A demonstrating on-target effects of drugs at the indicated doses. **B.** Immunoblotting (A375, MIA PaCa-2) and cell viability measures as assessed by crystal violet staining of cells in clonogenic assay plates (A375, A549), Cell Titer Glo (CTG) (A375, A549, MIA PaCa-2, MOLM13) or cell counts (HCC827) following treatment with vehicle (DMSO) or indicated targeted therapy for the indicated periods of time (24h for cell counts in HCC827). For clonogenic assay, cells were plated at the following densities: A375-1000 cells/well, A549-1000 cells/well. N=3 for cell viability experiments, where the mean ± S.E.M is plotted. *P* values were determined using unpaired, two-tailed Student’s *t*-tests. **C.** Cell viability as assessed by crystal violet staining of cells in clonogenic assay plates for GR4 cells (plated at 1500 cells/well), as well as Cell Titer Glo (CTG) following treatment with vehicle (DMSO) or gefitinib for the indicated periods of time. N=3 for cell viability experiments, where the mean ± S.E.M is plotted. *P* values were determined using unpaired, two-tailed Student’s *t*-tests.

**Figure S2:** **Additional** **characterization of EGFR inhibitor-induced ATM pathway activation.**  **A.** Immunoblotting of various DNA damage response markers in HCC827 cells following 24h treatment with gefitinib at the indicated doses. **B.** Immunoblot of PC9 cells treated with increasing concentrations of the third-generation EGFR inhibitor osimertinib for 24h, alongside Annexin V^+^ staining (normalized to DMSO vehicle control) in drug treated populations of PC9 cells at 24h. N=3 for Annexin V^+^ staining experiments, where the mean ± S.E.M is plotted. *P* values were determined using unpaired, two-tailed Student’s *t*-tests. **C.** Immunoblot of PC9 cells treated with vehicle control (DMSO) or EGFR inhibitor gefitinib (100 nM) for 24h, followed by drug removal and collection at 24h intervals. **D.** Immunoblot of shScramble or shEGFR cells following treatment with vehicle or 100 nM gefitinib for 24h. **E.** Immunoblot of PC9 cells treated with increasing concentrations of gefitinib, probing for additional members of DNA damage response pathways. **F.** Immunoblot confirming CRISPR/Cas9-mediated knockdown of BIM and RNAi-mediated knockdown of BAK and BAX in PC9 cells. **G.** Immunoblot of sgCTRL or sgBIM-1 cells following 24h treatment with vehicle or 100 nM gefitinib. **H.** Immunoblot confirmation of CRISPR/Cas9-mediated knockdown of caspase 3, 7, or 3+7 in PC9 and HCC827 cells. **I.** Immunoblot of HCC827 cells following CRISPR/Cas9-mediated knockdown of caspase 3, 7, or 3+7, post 24h vehicle or gefitinib (100 nM) treatment. **J.** Immunoblot of sgCTRL or sgCASP3/7 cells following 24h treatment with vehicle or 100 nM gefitinib. **K.** Immunoblot of cyclohexmide treated PC9 cells in the presence or absence of DMSO or gefitinib (100 nM) for 0-5 hours. **L.** Immunoblot confirmation of CRISPR/Cas9-mediated knockdown of CAD in EGFR inhibitor-sensitive PC9 cells and EGFR inhibitor-resistant PC9R and GR4 cells. **M.** Immunoblot of cells with CRISPR/Cas9-mediated knockdown of CAD (or non-targeting control CRISPR, sgCTRL) following treatment with vehicle or gefitinib (100 nM) for 24h. **N.** Bar graph quantification of extent tail moment (a.u.) from neutral comet assay performed in PC9 cells with or without the presence of CAD, following treatment with vehicle or 100 nM gefitinib for 24h. N=503 for sgCTRL+DMSO, N=704 for sgCTRL+Gefitinib, N=589 for sgCAD+DMSO, and N=664 for sgCAD+Gefitinib. The mean ± S.E.M is plotted. *P* values were determined using unpaired, two-tailed Student’s *t*-tests. **** refers to P<0.0001.

**O.** Confocal microscopy images of Rad51 loading assay in PC9 cells following treatment with vehicle (DMSO) or the combination of gefitinib (100 nM) and AZD0156 (1.5 μM) for 24h. **P.** Quantification of images in (O), with inclusion of data presented in Figure 2L. N=8 for DMSO treatment, N=3 for gefitinib treatment, N=3 for gefitinib+QVD and N=8 for gefitinib+AZD0156. The mean ± S.E.M is plotted. *P* values were determined using unpaired, two-tailed Student’s *t*-tests. * refers to P<0.05, ** refers to P<0.01, *** refers to P<0.001.

**Figure S3: Additional characterization of the effects of DNA damage response inhibitors on survival and growth of oncogene-driven cancer cell lines**: **A**. Immunoblot from PC9, PC9R, GR4 and WZR12 cells treated with the indicated doses of the EGFR inhibitor gefitinib for 24h. **B.** Immunoblotting of PC9R cells treated with vehicle (DMSO), gefitinib (100 nM), AZD0156 (1.5 μM) or the combination of gefitinib and AZD0156 for 72 hours. **C.** Annexin V^+^ staining (normalized to DMSO vehicle control) in drug treated populations of EGFR inhibitor-resistant PC9R cells after 72 hours of drug treatment (100 nM gefitinib and/or 1.5 μM AZD0156). N=3 for Annexin V^+^ staining experiment, where the mean ± S.E.M is plotted. *P* values were determined using unpaired, two-tailed Student’s *t*-tests, where *** refers to P<0.001. **D.** Immunoblotting of drug-sensitive (PC9) and drug-resistant (DTPs cultured in the presence of gefitinib or osimertinib, respectively) cells for baseline amounts of p-ATM. **E.** Immunoblot of cell fractionation experiments in PC9 cells and PC9-DTP cells, derived following serial gefitinib treatment as previously described. (WCL, whole cell lysate) **F.** Estimated cell number during long-term time-to-progression (TTP) assay of HCC827, MIA PaCa-2 and A549 cells treated with the cognate targeted therapies, AZD0156, or the combinations. N=3 for TTP experiment, where the mean ± S.E.M is plotted. **G.** Immunoblot of HCC827, MIA PaCa-2 and A549 cells treated with the cognate targeted therapies (100 nM gefitinib or 100 nM SCH772984) or AZD0156 (1.5 μM). **H.** Immunoblotting of various DNA damage response markers in MGH119 cells following 24 treatment with increasing concentrations of EGFR inhibitors gefitinib or osimertinib. **I.** Cell viability, as assessed through the percentage of surviving cells (normalized to gefitinib-only treated), of MGH119 drug-tolerant persisters (DTPs) following 4-day treatment with single-agent gefitinib (100 nM), AZD0156 (1.5 μM), or the combination. N=3 for DTP cell viability experiment, where the mean ± S.E.M is plotted. *P* values were determined using unpaired, two-tailed Student’s *t*-tests, where * refers to P<0.05. **J.** Cell viability in the indicated drug treatment conditions in ERK inhibitor-sensitive cells. N=3 for cell viability experiment, where the mean ± S.E.M is plotted. *P* values were determined using unpaired, two-tailed Student’s *t*-tests, where ** refers to P<0.01. **K.** Immunoblot of *KRAS*-mutant cell lines from (J) treated with the indicated doses of gefitinib for 24h. **L.** Immunoblot of PC9 cells following 24h drug treatment with or without 100 nM gefitinib in the presence (shScramble) or absence of ATM (shATM). **M.** Immunoblot of PC9 cells following 24h drug treatment with the indicated concentrations of vehicle, gefitinib or olaparib. **N.** Cell viability after three day treatment in the indicated drug treatment conditions in EGFR inhibitor-sensitive (PC9) and -resistant (PC9R, GR4) cells. N=3 for cell viability experiment, where the mean ± S.E.M is plotted. *P* values were determined using unpaired, two-tailed Student’s *t*-tests, where ** refers to P<0.01 and *** refers to P<0.001. **O.** Cell viability after three day incubation in the indicated drug treatment conditions in EGFR inhibitor-sensitive (PC9) and -resistant (WZR12) cells. N=3 for cell viability experiment, where the mean ± S.E.M is plotted. *P* values were determined using unpaired, two-tailed Student’s *t*-tests, where *** refers to P<0.001 and **** refers to P<0.0001. **P.** Estimated cell number during long-term TTP assay of PC9 cells treated with gefitinib, olaparib, or the combination. N=3 for TTP experiment, where the mean ± S.E.M is plotted. **Q.** Cell viability in the indicated drug treatment conditions in EGFR inhibitor-sensitive (PC9) and -resistant (GR4) cells with or without CAD presence (sgCTRL or sgCAD, respectively). N=3 for cell viability experiment, where the mean ± S.E.M is plotted. *P* values were determined using unpaired, two-tailed Student’s *t*-tests, where ** refers to P<0.01 and *** refers to P<0.001.


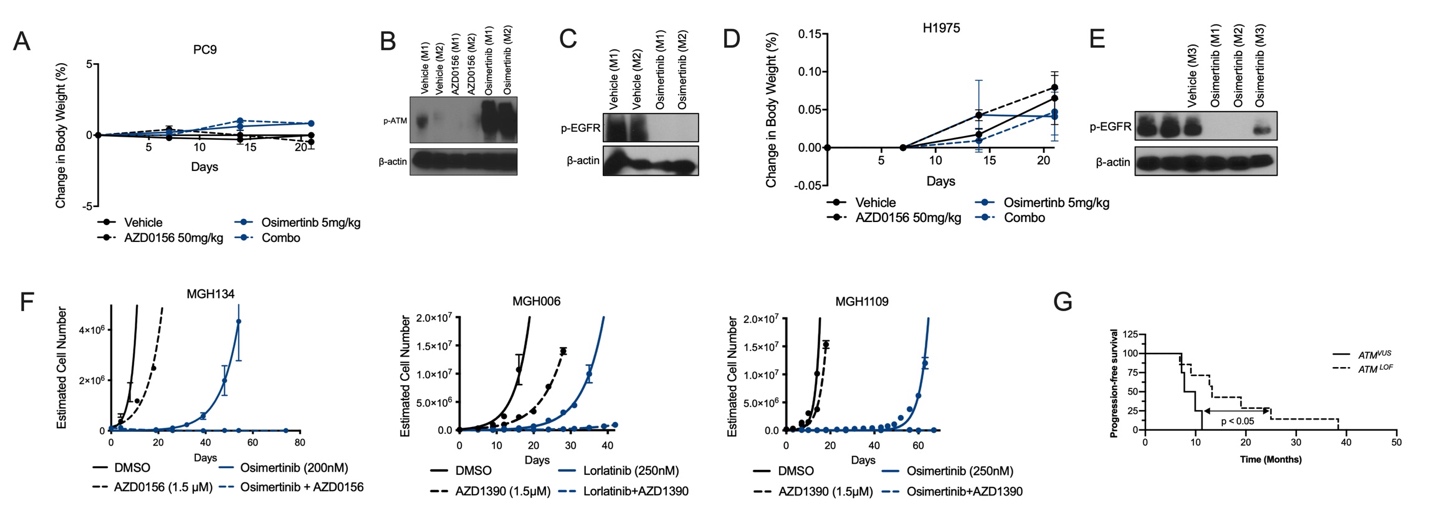


**Figure S4: Further characterization of mouse xenograft studies combining EGFR and ATM inhibitors**: **A.**Mean changes in body weight for *n*=5 mice per group of PC9 xenograft-bearing mice treated with vehicle, AZD0156, Osimertinib, or the combination. **B**and **C.**Immunoblotting of lysates from PC9 xenografts harvested immediately following treatment with vehicle, AZD0156, or osimertinib (2 mice shown per treatment group) for 5 days. **D.**Mean changes in body weight for *n*=5 mice per group of H1975 xenograft-bearing mice treated with vehicle, AZD0156, Osimertinib, or the combination. **E.**Immunoblotting of lysates from H1975 xenografts following treatment with vehicle or osimertinib (3 mice shown per treatment group). **F.**Estimated cell number during long-term time-to-progression (TTP) assay of MGH134, MGH006, and MGH1109 cells treated with the cognate targeted therapies, AZD0156, or the combinations. N=3 for TTP experiment, where the mean ± S.E.M is plotted. **G.**Kaplan-Meier curve indicating progression-free survival of patients with *EGFR*-mutant, *ATM*loss-of-function NSCLC versus *EGFR*-mutant, *ATM*variant of unknown significance NSCLC, in each case treated in the first line with the EGFR inhibitor erlotinib.
